# Teledentistry: a new oral care delivery tool among Indian dental professionals – a questionnaire study

**DOI:** 10.12688/f1000research.122058.1

**Published:** 2022-06-16

**Authors:** Kishan Paul Raja, Aindrila Pal, Sangeeta Umesh Nayak, Keshava Pai, Ramya Shenoy

**Affiliations:** 1 Periodontology, Manipal College of Dental Sciences, Mangalore Manipal Academy of Higher Education, Manipal, Karnataka, India; 2 Psychiatry, Kasturba Medical College, Mangalore Manipal Academy of Higher Education, Karnataka, India; 3 Public Health Dentistry, Manipal College of Dental Sciences, Mangalore Manipal Academy of Higher Education, Manipal, Karnataka, India

**Keywords:** Tele dentistry, Telemedicine, Health education, Oral care in rural area

## Abstract

**Background:** The sudden massive spread of coronavirus disease 2019 (COVID-19) has led to a major public health emergency and changed the scene of the health care globally. During the COVID-19 pandemic, most dental treatment procedures were considered as major sources of infection transmission. Thus, the current survey aimed at evaluating knowledge, awareness and attitude of dental professional of India towards teledentistry.

**Methods**: A pre structured questionnaire was framed and distributed among 600 dental professionals of India using email, WhatsApp and other social media modes.  SPSS version 17 was used for data analysis. The Chi-Square, student t test and one way ANOVA test were applied to assess the association between qualification, type of practice and participant response.  Logistic regression analysis was also carried out.

**Results: **In total, 431 dental professionals completed the survey. Overall, 94.7% of dental professionals were aware about teledentistry and agreed it is useful in fulfilling needs of the community at great amount. A statistically significant difference was found for questions related to the application of teledentistry for all branches, whether it is a good tool to assess oral hygiene in remote areas and its usefulness in training in primary health care centre when comparison was done among dental professionals and specialist in different branches of dentistry.

**Conclusions:** Within the limits of the study, findings show that study participants exhibited good knowledge and awareness regarding teledentistry. The participants exhibited positive attitude towards teledentistry but at the same time expressed the uncertainty in challenges which they may face in teledentistry. Lack of training, advanced infrastructure, good connectivity and network are main issues they were concerned about. The other important point of concern is many participants felt teledentistry cannot be applied for all branches of dentistry. Future research should focus on this aspect of teledentistry.

## Introduction

Computers and tele-communications are playing an important role in health care. Dental manpower shortages, remoteness, funding challenges, reduction of cost and technical developments, have led to increased awareness in the utilisation of telemedicine services. ^1^

The term “telehealth” and “e-health” came into reality because of advances in communication technologies and use of electronic information in health care services at a distance. ^1^^,^^2^

Telemedicine is a fragment of telehealth, and makes use of communication networks to deliver healthcare services and medical education from one geographical location to another. Teledentistry deals specifically with dental health and related issues, and is a combination of digital/telecommunication technology and dentistry. The use of teledentistry and its application in oral health services is of utmost importance in rural and distant locations. With the help of teledentistry one can recognize high risk populations, arrange referral to a dental surgeon or specialist, and encourage locally based treatment. Together all these leads to reduction in waiting period, unnecessary travel and loss of productivity. ^2^^,^^3^

The sudden and rapid spread of coronavirus disease 2019 (COVID-19) caused an unrivalled public health crisis which has altered the landscape of the health system globally, and thus teledentistry gained popularity during the COVID-19 pandemic. During this time, dental clinics and medical facilities were considered as spots of cross infection. A majority of dental treatment procedures deal with saliva, aerosol and droplet production which were considered as major sources of infection transmission. It was thus only emergency treatment that was permitted during the pandemic. This led to subsequent delay in diagnosis and management of dental diseases which caused devastating consequences in oral health conditions. ^3^^,^^4^

To overcome this, teledentistry consultation was encouraged so that with minimum contact between dentists and patients, the problems were addressed and both dentist and patient were benefited too. ^4^

In today’s era, a strong communication between dental professionals and patients can be achieved using high-speed mobile data, and good internet connectivity. The term “teledentistry,” is a mode of providing dental care across different regions. It has evolved with the use of smartphones, laptops, and various video conferencing software applications. It has totally reformed the traditional practice as it encourages a virtual mode of consultations and follow-up instead of direct one-to-one consultation. ^5^^,^^6^

In the management of patients with dental emergencies teledentistry can be of great help for patients. It can be delivered in two ways:

(i) Real-time consultation
(ii) Store and forward

Real-time consultation comprises a video conference connecting dentist and the patient, at different locations. During this time the dental professional can discuss with the patient about their problems and see their problems. The advanced telecommunication tools and high-speed internet connections makes this possible. ^7^^,^^8^

In the store and forward method, the telecommunication equipment is used to collect case material involving the clinical information and static images (clinical/radiographic/laboratory) and is stored. Then for consultant opinion and treatment planning this stored data is utilized. Thus in a quicker and cost effective manner treatment can be delivered to the targeted population. ^7^^,^^9^

Remote and new health care workers can utilize teledentistry as a strategy to support rural populations. It makes it possible to increase access to, and provision of oral care in rural and undeveloped areas. ^2^ Higher degree of acceptance for tele dentistry is seen among clinicians and patients compared to traditional direct consultation. ^5^^,^^6^^,^^7^ Very few studies have been done in this aspect in the Indian population. ^1^^,^^10^ Thus the current research aims at assessing the knowledge, awareness, and attitude regarding teledentistry among dental professionals of India.

## Methods

### Study design

The present survey used an online pre structured questionnaire among the dental professionals in India.

### Ethical considerations

The permission from the Institutional Ethics and Review Board of the Manipal College of Dental Sciences, Mangalore. India (protocol no: 21016) was obtained prior to commencement of the research. All participants gave informed consent through the Google form.

### Sample

The sample size was calculated using statistical software, considering 95% confidence interval, and an assuming 50% level of knowledge on teledentistry and its usefulness. The estimated sample size was 431 participants.

*Inclusion criteria and Exclusion criteria*

All the dental professionals whose dental degree was recognized by the Indian regulatory body and willing to participate in the survey by giving the consent were included in the study.

The dental students and interns whose internship is not complete or degree is not registered to governing body during the survey period were excluded.

### Questionnaire

Based on the available previous data the questionnaire was formulated. A preliminary pilot study was conducted among 40 dental professionals and this data were not included during the survey. The survey tool was validated.

The survey explained the objectives of the current research and the participants were asked to provide inform consent if there are willing to participate in the survey. A set of 20 questions were divided into three sections. The demographic information such as name, age, qualification, type of practices, year of experience were recorded in first section of the survey.

The knowledge and awareness regarding teledentistry was assessed in the second section using eight questions in that aspect. In the third section dental professional’s attitude was evaluated using five-point Likert scale. The two extremes of the Likert scale were strongly agree = 1 and strongly disagree = 5. The tool can be found as *Extended data.* ^22^

### Procedure

An electronic form - A google form with study information, informed consent and questionnaire was developed. The study team identified Email, WhatsApp, and Instagram groups of practicing dentists in India. The sharing of the questionnaire was done through the assigned social media platforms to a randomly selected sample of dental professionals in India (n = 600). This step helped us to control the potential selection bias. The data was collected from July 2021 to October 2021, and the survey was stated closed by November 2021.Two reminders were sent in an interval of two weeks to all the selected candidates.

### Statistical analysis

The SPSS statistical package was used for data analysis (IBM SPSS Statistics for Windows, Version 17.0, Released 2011, IBM Corp., Armonk, NY, USA, RRID:SCR_016479). Descriptive statistics were tabulated. Chi-Square, student’s t test and one way ANOVA tests were applied to assess the association between qualification, type of practice and participant response. Logistic regression analysis was also carried out. Professional qualification was kept as the dependent variable and questions (1-8) pertaining to knowledge of teledentistry were kept as independent variables. A p-value < 0.05 was considered significant.

## Results

**Figure 1.**
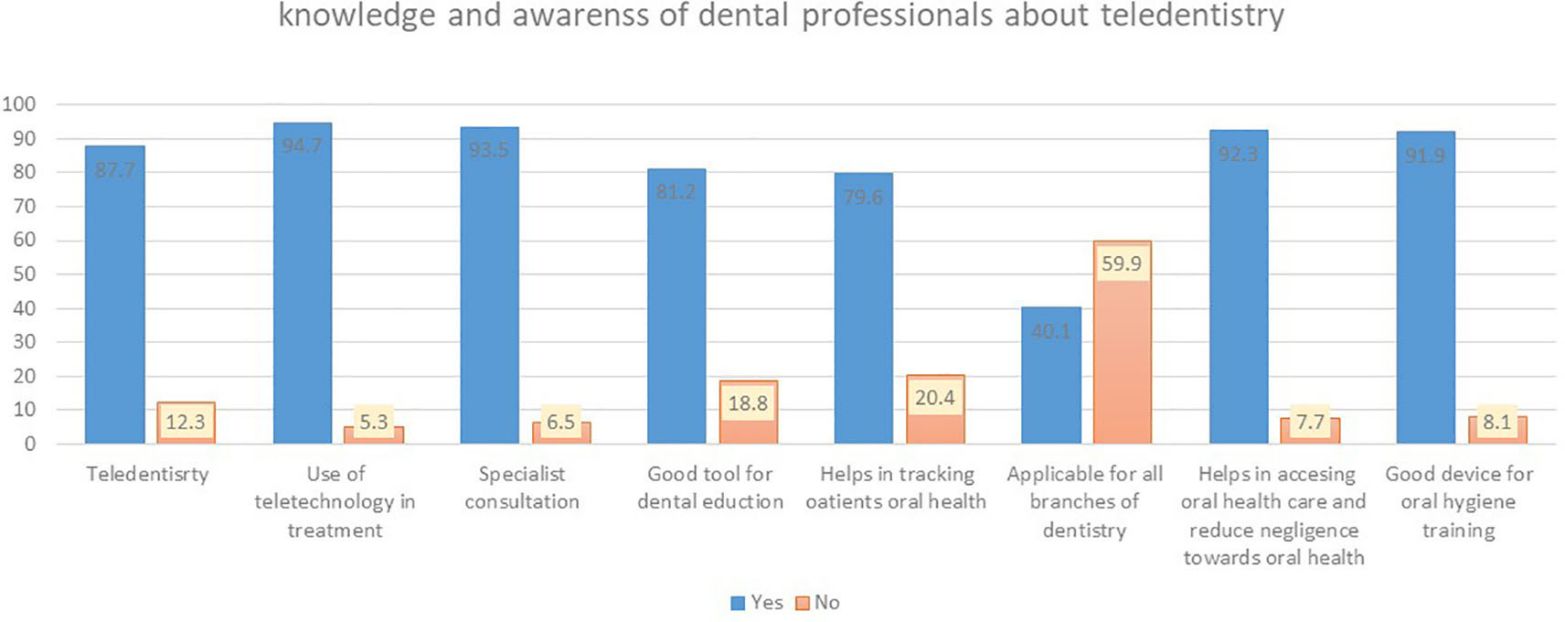
Knowledge and awareness of dental professionals about teledentistry.

In total, 600 google forms were emailed to dental surgeons all around India. Among which 431 complete filled forms we received. ^22^ Out of 431 participants, 378 (87.7%) were aware of the term “teledentistry” and 408 (94.7%) were aware of the use of teledentistry, its method of use and its usefulness in dental practice. 403 (93.5%) study participants felt teledentistry is useful in seeking opinion about disease diagnosis with a specialist. 349 (81.2%) study participants felt tele-dentistry can be a good educational tool. This can be used to create awareness and educate the primary care dental professionals in an effective way. 343 (79.6%) study participants felt teledentistry can be a good way to track the oral health of the patient. 258 (59.9%) study participants felt tele dentistry is difficult or challenging to apply to all branches of dentistry. Majority of study participants felt teledentistry can be used as effective tool in teaching oral care, to create awareness among health professionals and patients and circulate the information regarding the access to information pertaining to oral hygiene facility ( Figure 1).

**Figure 2.**
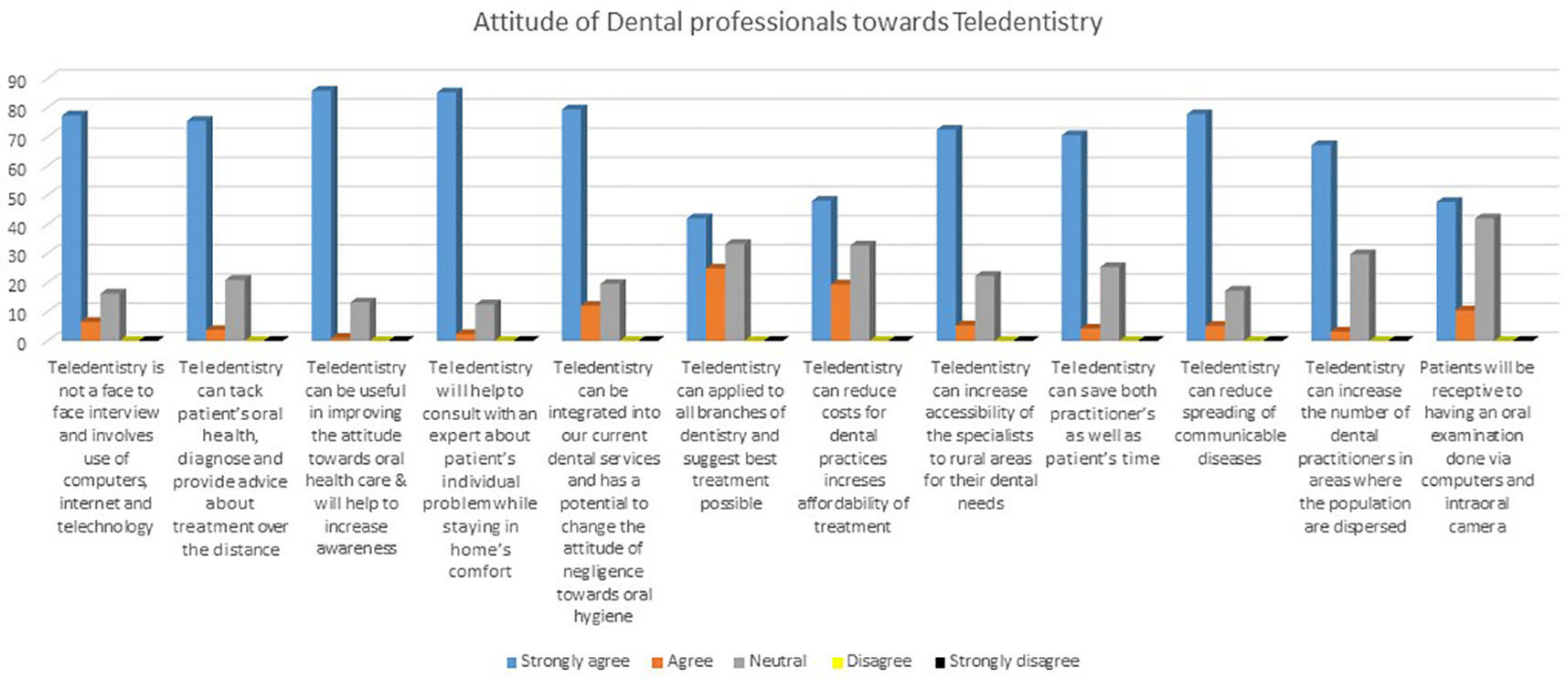
Attitude of dental professionals towards teledentistry.

A questionnaire (set of 12 questions) was used to access the attitude towards tele-dentistry ( Figure 2). A mixed response was obtained from the study participants. The responses ranged from strongly agree, agree and neutral.

Categorization of different qualifications was done using student’s t test. Out of 431 participants 281 participants were dental surgeons with a BDS degree and 150 participants were specialist in various branches of dentistry. The distribution was statistically significant with a P value of 0.036. One way ANOVA showed uniform distribution among the type of practice (private, academician or both) ( Table 1).

**Table 1.** One way ANOVA test for type of dental practice and response by the study participants.

	Sum of squares	df	Mean square	F	Sig.
Between Groups	119.667	2	59.834	2.061	.129
Within Groups	12426.328	428	29.033		
Total	12545.995	430			

The chi square test and logistic regression analysis showed a statistically significant difference among dental surgeons (BDS) and specialists (MDS) regarding teledentistry as an educational tool to train dental professionals in primary care centres in remove/rural areas (question no 4), it is an effective tool in monitoring patient’s oral health (question no 5) and can be applied to all branches of dentistry (question 6). A statistically significant difference with p value of 0.005 was found for the questions regarding teledentistry as an effective tool for oral hygiene training (question8) ( Tables 2, 3 & 4).

**Table 2.** Chi square test for qualification of the study participant and response regarding teledentistry.

S.No	Question		Yes-	NO	Chi square value	Df	P value
			Number of participants				
			N	N			
Q1	Have you come across the term- Tele-dentistry?	BDS	241	40	2.812	1	0.061
		MDS	137	13			
Q2	Does Tele-dentistry revolve around the practice of use of digital/telecommunication technologies in diagnosing and providing advice about treatment over a distance?	BDS	264	17	0.813	1	0.253
		MDS	144	6			
Q3	Does Tele-dentistry assist in arranging consultation with a specialist about patient’s problem?	BDS	260	21	1.268	1	0.179
		MDS	143	7			
Q4	Is Tele-dentistry good for dental education over internet and for training primary health-care dentists?	BDS	216	65	9.957	1	**0.001** *
		MDS	134	16			
Q5	Does Tele-dentistry help to track patient’s oral health?	BDS	207	74	17.396	1	**0.00** *
		MDS	136	14			
Q6	Can Tele-dentistry be applied to all the branches of dentistry?	BDS	90	191	22.106	1	**0.00** *
		MDS	83	67			
Q7	Is Tele-dentistry beneficial in improving the access to oral health care and decreases neglect towards oral health?	BDS	258	23	0.319	1	0.360
		MDS	140	`10			
Q8	Do you agree that Tele-dentistry is a worthy tool for oral hygiene training?	BDS	260	21	0.453	1	0.309
		MDS	136	14			

BDS - Bachelor of Dental Surgery, MDS - Master of Dental Surgery.

* P value stastically significant ≥ 0.005, df - degrees of freedom.

**Table 3.** Type of dental practice and response regarding teledentistry.

S.No	Question		Yes	No	Chi square value	df	P value
			Number of participants				
			N	N			
Q1	Have you come across the term- Tele-dentistry?	Private	143	17	2.236	2	0.327
		Academics	173	23			
		Both	62	13			
Q2	Does Tele-dentistry revolve around the practice of use of digital/telecommunication technologies in diagnosing and providing advice about treatment over a distance?	Private	154	6	1.880	2	0.391
		Academics	185	11			
		Both	69	6			
Q3	Does Tele-dentistry assist in arranging consultation with a specialist about patient’s problem?	Private	146	14	2.512	2	0.285
		Academics	187	9			
		Both	70	5			
Q4	Is Tele-dentistry good for dental education over internet and for training primary health-care dentists?	Private	122	38	5.257	2	0.072
		Academics	168	28			
		Both	60	15			
Q5	Does Tele-dentistry help to track patient’s oral health?	Private	126	34	0.588	2	0.745
		Academics	159	37			
		Both	58	17			
Q6	Can Tele-dentistry be applied to all the branches of dentistry?	Private	50	110	10.318	2	**0.006** *
		Academics	94	102			
		Both	29	46			
Q7	Is Tele-dentistry beneficial in improving the access to oral health care and decreases neglect towards oral health?	Private	145	15	3.438	2	0.179
		Academics	186	10			
		Both	67	8			
Q8	Do you agree that Tele-dentistry is a worthy tool for oral hygiene training?	Private	149	11	7.622	2	**0.022** *
		Academics	184	12			
		Both	63	12			

* P value stastically significant ≥ 0.005, df - degrees of freedom.

**Table 4.** Logistic regression analysis qualification as dependent variable and knowledge of teledentistry as independent variable.

	B	S.E.	Wald	df	Sig.	Exp(B)	95% C.I.for EXP(B)	
Step 1 ^a^							Lower	Upper
Q1(1)	.143	.408	.124	1	.725	1.154	.519	2.566
Q2(1)	.487	.564	.748	1	.387	1.628	.539	4.915
Q3(1)	-.242	.553	.191	1	.662	.785	.266	2.321
Q4(1)	.626	.365	2.945	1	.086	1.870	.915	3.823
Q5(1)	1.420	.413	11.821	1	**.001** *	4.138	1.841	9.297
Q6(1)	.737	.234	9.915	1	**.002** *	2.090	1.321	3.306
Q7(1)	-.440	.494	.796	1	.372	.644	.245	1.694
Q8(1)	-1.454	.516	7.926	1	**.005** *	.234	.085	.643
TOTAL SCORE	.010	.024	.164	1	.685	1.010	.964	1.058
Constant	-1.457	.967	2.268	1	.132	.233		

S.E. - Standard Error, C.I. - Confidence interval, df - Degrees of Freedom.

^a^ Variable(s) entered on step 1: Q1, Q2, Q3, Q4, Q5, Q6, Q7, Q8, TOTAL SCORE.

* P value stastically significant ≥ 0.005.

## Discussion

With the advancement in technology, the world is changing in all directions. Dental practitioners also should be aware about the changing trends in their field. They should be updated with knowledge which will assist them to face the challenges in their filed. They should be updated with knowledge and skills so that they will be able to fulfil the necessary oral health care and the ongoing requirements of their community.

Teledentistry is a mode of delivery of oral care in which dental care is provided to the patient without direct contact or with minimal contact. This all is possible because of usage information technology and high speed internet facility.

Since there are limited challenges in clinical examination of the oral cavity, intraoral photographing can be carried out with little knowledge or guidance by patients or by -standers. ^11^

Although tele dentistry is not a new treatment modality, the COVID-19 pandemic did raise the attention of the dental community towards teledentistry and the important role it can play as a tool for the delivery of a wide range of dental services in near distant places with zero risk of infection. ^12^

In the present study a large section of respondents did agree that teledentistry would help to minimise the waiting time period. They were also of the opinion that teledentistry can improve the interaction among colleagues to seek advice regarding patients. Teledentistry is also believed to manage patient referral effectively and in providing a safe atmosphere for practicing dentistry.

In the present study 94.7% study participants were aware about teledentistry which is in contrast to earlier studies. ^1^^,^^12^^–^^14^ In the earlier studies it was opined that a smaller number of dentists were aware of teledentistry because of a lack of knowledge in this field. This was mainly attributed to having no didactic and practical training in this field as it was not a part of the dental curricula, and also a lack of continuous education programs on teledentistry. Hence, integrating teledentistry in the undergraduate and postgraduate curricula and organising lectures and hands on workshops as a part of continuing dental education is of paramount importance to fill the knowledge gap. In the present study the participants were found to be aware about the concept of teledentistry. This is probably due to the involvement of technology in dental curriculum (paperless exams, online teaching, webinars by dental regulatory bodies (dental council of India). Nowadays in the health care system, application of computers and internet has become common. In the field of teledentistry a link is created between dental health care providers and patients so that there is improvement in communication, exchange of health-related information and access to dental care dental health records in different locations. ^15^^,^^16^ In the present study, 93.5% of participants felt teledentistry is useful in seeking opinion about disease diagnosis with a specialist. 81.2% study participants felt tele-dentistry can be a good educational tool and this is similar to earlier studies. ^12^^,^^17^ The study participants showed a positive attitude towards teledentistry and this was similar to earlier studies. ^12^^,^^17^ It was seen that 59.9% study participants felt that teledentistry is difficult or challenging to apply to all branches of dentistry, and this was in accordance with earlier studies. ^12^^,^^17^ Disagreement among dentists exists regarding the accuracy and reliability of diagnosis through teledentistry. ^12^^,^^18^^–^^20^ The reliability of examination via the application of teledentistry should be assessed and supported by more research directed in this direction.

About 60% of dentists accepted the potential helpfulness of teledentistry for patients. In addition, its usefulness among patients in rural and remote areas was expressed by more than 70% of the dentists. The benefit of teledentistry in terms of patient education, patient monitoring and a reduction of the need to travel to the dental clinic was expressed by 70% of the responding dentists. ^1^ A study by Fernandez C E in 2021, also stated teledentistry as an effective means for oral health improvement in terms of prevention and promotion. ^21^ The application of teledentistry to help detect and diagnose dental caries has also been reported in a systematic review of 10 studies. ^18^

Limitation of the study: Around 169 forms (39%) were not included in the data analysis due to lack of clarity and incomplete details. The study included both practitioners and academicians which may be some amount of impact on their knowledge regarding teledentistry.

## Conclusion

Within the limits of the study, participants exhibited good knowledge and awareness regarding teledentistry. The participants exhibited positive attitudes towards teledentistry but at the same time expressed the uncertainty in challenges which they may face in teledentistry. Lack of training, advanced infra structure, good connectivity and network are main issues which they were concerned about. The other important point of concern is many participants felt teledentistry cannot be applied for all branches of dentistry. Future research should focus on this aspect of teledentistry.

## Data availability

### Underlying data

Figshare: Teledentistry: New Oral Care Delivery Tool among Indian Dental Professionals –A questionnaire Study. https://doi.org/10.6084/m9.figshare.19915021. ^22^

This project contains the following underlying data:

- teledentistry- data.ods

### Extended data

Figshare: Teledentistry: New Oral Care Delivery Tool among Indian Dental Professionals –A questionnaire Study. https://doi.org/10.6084/m9.figshare.19915021. ^22^

This project contains the following extended data:

- Case proforma.docx (questionnaire)

Data are available under the terms of the Creative Commons Zero “No rights reserved” data waiver (CC0 1.0 Public domain dedication).
