# Supplementary material for: Teledentistry: a new oral care delivery tool among Indian dental professionals – a questionnaire study
Source: F1000Res. 2022 Jun 16;11:666. [Version 1] doi: 10.12688/f1000research.122058.1 (PMC9471219; doi:10.12688/f1000research.122058.1)
Supplement: Peer review history

# Peer review: Reviewer response for version 1

**DOI:** 10.5256/f1000research.133996.r144019

**Published:** 2022-09-13

**Authors:** Vidya Shenoy

**Affiliations:** 1 Department of Prosthodontics, AJ Institute of Dental Sciences, Mangalore, India

The current survey aimed at evaluating knowledge, awareness and attitude of dental professional of India towards teledentistry. In total, 431 dental professionals completed the survey. Overall, 94.7% of dental professionals were aware of teledentistry and agreed it is useful in fulfilling the needs of the community at great amount. Within the limits of the study, findings show that study participants exhibited good knowledge and awareness regarding teledentistry. The participants exhibited a positive attitude towards teledentistry but at the same time expressed the uncertainty in challenges that they may face in teledentistry. Lack of training, advanced infrastructure, good connectivity and network are the main issues they were concerned about. The other important point of concern is many participants felt teledentistry cannot be applied to all branches of dentistry. Future research should focus on this aspect of teledentistry.

 Work is clearly and accurately presented with a technically sound design which will help the readers in reproducibility. Conclusions are drawn based on the results obtained.

Is the work clearly and accurately presented and does it cite the current literature?

Yes

If applicable, is the statistical analysis and its interpretation appropriate?

I cannot comment. A qualified statistician is required.

Are all the source data underlying the results available to ensure full reproducibility?

Yes

Is the study design appropriate and is the work technically sound?

Yes

Are the conclusions drawn adequately supported by the results?

Yes

Are sufficient details of methods and analysis provided to allow replication by others?

Yes

Reviewer Expertise:

Dentistry, oral health, geriatric oral health

I confirm that I have read this submission and believe that I have an appropriate level of expertise to confirm that it is of an acceptable scientific standard.

# Peer review: Reviewer response for version 1

**DOI:** 10.5256/f1000research.133996.r148514

**Published:** 2022-09-13

**Authors:** Harsh Priya

**Affiliations:** 1 Division of Public Health Dentistry, Centre for Dental Education and Research, All India Institute of Medical Sciences, New Delhi, Delhi, India

This is a good initiative to utilize the social media platform for evaluating knowledge, awareness and attitude of dentists towards teledentistry.

 A few concerns regarding the questionnaire, if the validity was assessed. An explanation regarding the content validity of the questionnaire will be appreciated.

 It would also be helpful to explain the online consent form a little more. Now, many studies will be performed online hence a clarification for readers would reinforce the procedure.

 Utilization of teledentistry in the primary health centres in rural areas should be elaborated on in the discussion. The preventive and public healthcare aspects may be discussed. This is a very important aspect here.

 How can the inclusion of practitioners and academicians change the results?

 Thank you

 Best Regards

 Harsh Priya

Is the work clearly and accurately presented and does it cite the current literature?

Yes

If applicable, is the statistical analysis and its interpretation appropriate?

Partly

Are all the source data underlying the results available to ensure full reproducibility?

Yes

Is the study design appropriate and is the work technically sound?

Yes

Are the conclusions drawn adequately supported by the results?

Yes

Are sufficient details of methods and analysis provided to allow replication by others?

Yes

Reviewer Expertise:

Public Health

I confirm that I have read this submission and believe that I have an appropriate level of expertise to confirm that it is of an acceptable scientific standard.

# Peer review: Reviewer response for version 1

**DOI:** 10.5256/f1000research.133996.r141715

**Published:** 2022-07-12

**Authors:** Melwin Mathew

**Affiliations:** 1 Periodontics and Oral Implantology, Manipal University College Malaysia, Melaka, Malaysia

The study has been conducted with the current issue faced by the community during the pandemic lockdown and when patients were unable to visit a clinic due to movement restrictions or fear of transmission of COVID-19 while visiting a medical/dental clinic. The study design is well structured and a well-balanced validated questionnaire has been used to assess the knowledge and attitude of dental practitioners towards teledentistry.

 In this study, a total of 431 participants responded out of 600 questionnaires sent out online, the awareness regarding the term "Teledentistry" and applications of teledentistry was highly significant among the participants however, the knowledge and awareness of applicability for all branches of dentistry in teledentistry was not significant as 59.9% answered "NO". 

 Meanwhile, assessing their attitude, the participants strongly agreed teledentistry was useful for improving patients' awareness and attitude toward oral health and providing the comfort of online consultation with a specialist. Apparently, the response was more neutral about patients' acceptance of online oral examinations using computers and intraoral cameras.

 Limitations of the study, that could be addressed in the future is, in assessing the practicality of the use of Teledentistry in rural, semi-urban and urban areas equally based on the accessibility of the internet by identifying participants in each of these areas and also assessing the agreement from the participants about setting up a large database to store the clinical data collected from teledentistry.

 This study has concluded by adequately supporting their results.

Is the work clearly and accurately presented and does it cite the current literature?

Yes

If applicable, is the statistical analysis and its interpretation appropriate?

I cannot comment. A qualified statistician is required.

Are all the source data underlying the results available to ensure full reproducibility?

Yes

Is the study design appropriate and is the work technically sound?

Yes

Are the conclusions drawn adequately supported by the results?

Yes

Are sufficient details of methods and analysis provided to allow replication by others?

Yes

Reviewer Expertise:

Dentistry, Periodontics, Dental Implants, Laser dentistry, Dental Radiology, Dental disinfection and decontamination, Practice management

I confirm that I have read this submission and believe that I have an appropriate level of expertise to confirm that it is of an acceptable scientific standard.
